# Ancillary Care: From Theory to Practice in International Clinical Research

**DOI:** 10.1093/phe/pht015

**Published:** 2013-06-13

**Authors:** Bridget Pratt, Deborah Zion, Khin Maung Lwin, Phaik Yeong Cheah, Francois Nosten, Bebe Loff

**Affiliations:** Monash University; Monash University; Shoklo Malaria Research Unit; Shoklo Malaria Research Unit; Mahidol-Oxford Tropical Medicine Research Unit; University of Oxford; Shoklo Malaria Research Unit; Mahidol-Oxford Tropical Medicine Research Unit; University of Oxford; Monash University

## Abstract

How international research might contribute to justice in global health has not been substantively addressed by bioethics. This article describes how the provision of ancillary care can link international *clinical* research to the reduction of global health disparities. It identifies the ancillary care obligations supported by a theory of global justice, showing that Jennifer Ruger’s health capability paradigm requires the delivery of ancillary care to trial participants for a limited subset of conditions that cause severe morbidity and mortality. Empirical research on the Shoklo Malaria Research Unit’s (SMRU) vivax malaria treatment trial was then undertaken to demonstrate whether and how these obligations might be upheld in a resource-poor setting. Our findings show that fulfilment of the ancillary care obligations is feasible where there is commitment from chief investigators and funders and is strongly facilitated by SMRU’s dual role as a research unit and medical non-governmental organization.

## Introduction

International health research partnerships were identified as a ‘powerful tool’ for advancing health in low- and middle-income countries (LMICs) and promoting global health equity by the Commission on Health Research for Development in 1990 (CHRD, 1990: xvii). Since then, the premise has been reiterated in World Health Organization reports and at global ministerial summits on health research (Ministerial Summit on Health Research, 2004; WHO Task Force on Health Systems Research, 2005; Global Ministerial Forum on Research for Health, 2008).

The idea that international health research should contribute to global health equity led to the identification of a new role for research ethics—linking research to advancing health and building research capacity in LMIC host communities. In 2000, Solomon Benatar and Peter Singer argued that ‘a new, proactive research ethics … must ultimately be concerned with reducing inequities in global health and achieving justice in health research and health care’ (Benatar and Singer, 2000: 826). Progress has been made towards this objective with the development of concepts (Benatar and Singer, 2010) such as:

- responsiveness to local health concerns in host communities and/or countries (NBAC, 2001; CIOMS, 2002; Nuffield Council on Bioethics, 2002; London, 2005; World Medical Association, 2008),
- ancillary care (i.e. health care that is not required for either the scientific validity of a study or redressing study-related harms) (Belsky and Richardson, 2004; Merritt *et al.*, 2010),
- post-trial benefits such as medical treatments or practices developed by a study (NBAC, 2001; CIOMS, 2002; Nuffield Council on Bioethics, 2002; Emanuel, 2008; Macklin, 2008; World Medical Association, 2008) and
- research capacity strengthening in host communities and/or countries (NBAC, 2001; CIOMS, 2002; UNAIDS/WHO, 2007; Meslin, 2008).

This article builds on earlier research that considers how, using such concepts, international clinical research should contribute to the reduction of global health inequalities. That research applied the principles of a theory of justice drawn from political philosophy to determine what is owed by external research actors from high-income countries to improve the health of individuals in LMICs. Jennifer Ruger’s health capability paradigm, a theory that extends the work of Amartya Sen and Martha Nussbaum, was relied upon because it has features that make it particularly suitable for deriving guidance on such matters (Pratt *et al.*, 2012a).^1^ In brief, the health capability paradigm demands that international clinical research target health conditions that are major contributors to host communities’ gap in health status from the optimal global level, be conducted in partnership with local researchers and build their capacity to conduct clinical research on diseases of local concern on their own (Pratt *et al.*, 2012a; Pratt and Loff, 2012). For the purposes of this article, it should be assumed that the research discussed meets these criteria.

Building on that work, our article derives ancillary care obligations from the health capability paradigm and discusses empirical research undertaken in order to describe how these obligations can be upheld. Developing robust guidance for ancillary care demands a normative model with the following features: a principled basis for determining that researchers have ancillary care obligations, specification of the content of these obligations and definition of the obligations’ upper and lower limits (Merritt, 2011). Thus far, a normative model has not been developed that relies on a theory of global justice to identify ancillary care obligations. The two normative models that have been proposed—the partial-entrustment model and the whole-person model—argue for the existence of special ancillary care duties for researchers (and sponsors) above general duties of rescue.^2^ The partial-entrustment model holds that special ancillary care duties derive from a morally significant feature of the researcher–participant relationship—the entrustment of aspects of participants’ health to researchers^3^—whereas the whole-person model considers such duties to be based on the moral significance of the researcher–participant relationship as a whole (Belsky and Richardson, 2004; Richardson, 2007; Dickert and Wendler, 2009).^4^ Efforts have subsequently been made to define the content of the duties supported by each model, particularly for researchers. A two-step framework has also been developed to assist with the identification of baseline ancillary care obligations derived from the duty of rescue (Merritt *et al.*, 2010). This framework can supplement either the partial-entrustment or whole-person model (Merritt, 2011).

Ancillary care obligations described by the partial-entrustment and whole-person models are not intended to connect international clinical research with global health equity. There has been minimal investigation into what guidance theories of global justice can offer on ancillary care provision in international clinical research. At most, duties to remedy global injustice have been used to ground broad ancillary care obligations for medical researchers (London, 2005). These ‘expansive arguments’ have been criticized for failing to identify why researchers should bear a greater burden for reducing global health inequities than other citizens of high-income countries. Richardson (2007: 1957) notes ‘general appeals to justice are difficult to translate into obligations incumbent on researchers in particular.’ It has further been asserted that an obligation to address *all* ancillary care needs encountered over the duration of international clinical trials would ‘strain budgets and monopolize the scarce time of trained personnel’ (Participants in the 2006 Georgetown University Workshop on the Ancillary-Care Obligations of Medical Researchers Working in Developing Countries, 2008: e90).

Hyder and Merritt (2009) state that debate is required about the precise ethical justification of ancillary care. In the philosophical analysis section of this article, we derive guidance on ancillary care from the health capability paradigm and briefly compare it to what is required by existing normative models. This supplements’ existing work by showing that a theory of justice is able to ground ancillary care obligations *and* allocate specific obligations of justice to external research actors from high-income countries, including governments, research funders, sponsors, and investigators. These ancillary care obligations do not require delivery of unlimited health care to participants during trials. Instead, the health capability paradigm supports an obligation to provide ancillary care for a limited subset of health conditions causing severe morbidity and mortality in host communities.

Constructing a normative model, however, is not sufficient to ensure obligations are met in the research setting in most cases. A recent consensus paper noted, ‘empirical research … about current ancillary-care practices, should be undertaken to inform the debate and the development of appropriate guidelines’ (Participants in the 2006 Georgetown University Workshop on the Ancillary-Care Obligations of Medical Researchers Working in Developing Countries, 2008: e90). To translate ethical requirements into practice, empirical investigation is needed to identify where ancillary care obligations are observed and to identify contextual factors that facilitate or obstruct adherence. The findings of empirical research can inform the development of practical guidance for research actors on how to uphold these obligations, which, in turn, will promote greater fulfilment (Pratt *et al.*, 2012b).

There has been some empirical research conducted that describes the provision of ancillary care in international research (Heise *et al.*, 2008; Taylor *et al.*, 2011). Of these studies, none has investigated the alignment of current practice with the guidance of a normative model, let alone provided information on *how* adherence to the model might be achieved. In the empirical section of this article, we describe the results of case study research that examined whether the ancillary care obligations supported by the health capability paradigm were upheld in the Shoklo Malaria Research Unit’s (SMRU) ongoing vivax malaria treatment trial. This was a retrospective application of a newly created ethical standard, as the vivax malaria treatment trial was not designed to adhere to the obligations we derived from the health capability paradigm. The data show that SMRU investigators not only met their ancillary care obligations but also provided health care beyond that required by the justice model described here. We identify the factors instrumental to SMRU investigators fulfiling their ancillary care obligations. We do not compare SMRU’s provision of care to other normative models, as our aim is to determine whether obligations linking ancillary care delivery to global justice can be met.

## Philosophical Analysis

### Understanding the Health Capability Paradigm

Jennifer Ruger’s health capability paradigm is both a theory of health justice *and* a framework for a system of global health governance founded upon shared ethical commitment to equity in health. As such, it operates concurrently at theoretical and practical levels. It seeks to establish new norms and to investigate how they might be applied using current global health architecture as the starting point.

The health capability paradigm establishes a universal obligation to efficiently reduce shortfall inequalities in individuals’ central health capabilities, particularly in countries where the shortfall from the optimal level is large (Ruger, 2010). Central health capabilities refer to individual ability and freedom to achieve certain health functionings (i.e. avoiding preventable morbidity and mortality). Reducing shortfall inequalities in health status for an individual or population refers to diminishing the gap in their health status from the optimal level (the highest level of health achieved worldwide in terms of life expectancy, disease prevalence and incidence and other variables). The paradigm envisions a shared health governance model where individuals, health goods and services providers and health-related institutions work together to reduce these shortfalls (Ruger, 2010).

Underlying this model of governance are shared moral values and ‘voluntary commitments’. Voluntary commitment is defined as the process through which the ethical norm of health equity is internalized at the collective and individual levels. Ruger (2009: 271) states ‘[o]nce individuals internalize these ethical commitments, they freely embrace them and obligate themselves to conform to them, sacrificing some of their resources and autonomy to be regulated and to distribute those resources to others.’ Voluntary commitments are the glue holding the system of shared health governance together, leading individuals and institutions to voluntarily make choices and take positive measures to ensure health disparities are reduced worldwide. Once the norm of health equity is internalized at the collective level, most actors will not have to be coerced to carry out their duties of justice, though regulatory mechanisms may be introduced to encourage duty fulfilment. Ruger describes how commitment to the public norm of health equity could be created by WHO and states (Ruger, 2009; Ruger and Yach, 2009). Her theory recognizes that this norm internalization will occur gradually over time, as health equity is not necessarily embraced by populations worldwide today.

Under the shared health governance model, actors within the health sector (which includes health research) work towards fulfiling the universal moral duty to reduce shortfall inequalities in health capabilities by upholding specific duties that are consistent with and arise from their role in society. Unlike the majority of cosmopolitan theories of justice, Ruger’s paradigm provides a principle for assigning particular duties to protect and maintain the central health capabilities to specific parties. Without such a second-stage principle, ‘it would be difficult, if not impossible, to allocate responsibilities among the multitudes and levels of institutions and actors’ (Ruger, 2009: 272). A theory of justice would offer little justification for why specific actors ought to act in particular ways. According to the functional requirements principle, duties are distributed to institutions or actors because the functions that they typically assume make them particularly capable of performing the duties (Ruger, 2009). The principle recognizes that institutions and individuals have respective roles in addressing health issues that make them the pragmatic choice to carry out certain duties of justice. Institutions and actors’ mandated roles and activities equip them with the skills, resources and authority to discharge the duties.

Individuals and institutions are allocated obligations of justice that are consistent with their functions *prior* to societies internalizing the norm of health equity (i.e. in the absence of voluntary commitments). As a result, until individuals and institutions within states embrace the norm, incentive measures such as government regulation and oversight will probably be required to ensure that they fulfil their obligations (Ruger, 2011).

### Grounding Ancillary Care Obligations for Research Actors

In accordance with the functional requirements principle, the primary responsibility for promoting health capabilities is allocated to states. National governments are in the best position to reduce the shortfall between their population’s health status and the optimal level. States must establish public health, health care and health research systems in order to ensure that their populations are able to obtain the goods and services necessary to guarantee central health capabilities—namely, public health goods and services; health care goods and services for prevention, diagnosis, treatment and rehabilitation; social support services; adequate nutrition and sanitary and safe living and working conditions (Ruger, 2010).

Where states do not reduce shortfalls in their citizens’ health capabilities from the optimal level, global actors have an obligation to assist such states, though states retain primary responsibility (Ruger, 2009). (As it is pertinent to our case study, we also take the position that where government persecution leads members of a state’s population to seek refuge in another country, the country of refuge acquires a secondary obligation to ensure those individuals’ basic capabilities such as health, though the state of origin retains the primary responsibility.) Global actors must *support and facilitate* state efforts to promote health, particularly where a population’s gap from the optimal health status is large, in accordance with their function. External research actors from high-income countries then have an obligation to perform international research in a manner that reduces global health disparities (Pratt *et al.* 2012a). We have previously described what this entails in terms of selecting research targets, capacity-building and post-trial benefits (Pratt and Loff, 2012). Here, we take the position that this obligation further includes providing ancillary care during research. Assisting with the delivery of health care where state health systems (public and private) are not doing so effectively can improve the health of the worst-off in LMICs. Research actors’ obligation to provide ancillary care further corresponds to their specific research role (i.e. funder, sponsor or researcher).

To conduct clinical research, investigators engage in repeated interactions with trial participants in a medical setting. As clinical researchers, they are likely to have the skills and resources to make a restricted contribution to address health conditions experienced by participants that are not dealt with by the local health system. The provision of health care unrelated to studies may be viewed as extraneous to the researchers’ role. However, physician-investigators will be especially capable of facilitating the delivery of a limited amount of care to study participants in LMICs that they would not otherwise receive. This includes care required to ensure a trial’s scientific validity and (ancillary) care that is not.

National governments, research funders and sponsors from high-income countries similarly acquire ancillary care obligations that align with their functions (Table 2).^5^ Governments are obligated to use their legislative powers to create a supportive policy environment for the provision of ancillary care. Research funders are obligated to finance the provision of ancillary care during trials. Funders acquire this obligation not simply because they are able to provide money but because they have a mandate to allocate their funds to research activities, including collateral benefits of research, and access to the channels to do so efficiently. Funding organizations are generally most able to spend their money in ways that are consistent with their missions. For example, Wellcome Trust does not support post-trial access to efficacious study interventions because doing so falls outside its remit as a *research* funder. However, it does support the provision of collateral benefits *during* research programs, including ancillary care, insofar as doing so does not adversely affect the local research environment (Wellcome Trust, 2009). Sponsoring institutions have a responsibility to support their researchers accurately identify the ancillary care needs that they are likely to encounter in particular overseas research settings prior to trials commencing. In instances where there is no longstanding collaboration or clear demographic information on disease burden, sponsors should assist researchers to liaise with health care providers in host districts or communities to gather information on the health conditions experienced by the local population.

At present, external research actors from high-income countries have ancillary care obligations that are grounded by their role in research. These obligations will eventually be strengthened through voluntary commitments (the process of norm internalization).^6^ Once individuals and institutions freely internalize a normative ethical commitment to health equity, they are obligated to act in ways that align with their commitment (Ruger, 2012). This reinforces/strengthens all function-related duties that are consistent with the norm, including the duty to fund and perform research that contributes to the reduction of shortfall inequalities in health in LMICs, which, in turn, demands (among other things) the provision of ancillary care. Thus, in time, the ancillary care obligations of research actors from high-income countries will be grounded in *both* their ‘voluntary commitment’ to changed norms and their function.

As a minimum, ancillary care obligations are owed to trial participants. In some trials, investigators may have substantial interaction with participants’ families. This may extend the obligations to them, but, in most situations, the claimants of care will be trial participants because they are directly part of the research enterprise. Under the health capability paradigm, actors are obligated to offer care to those in need who they are especially able to help in light of their functions. External research actors are best positioned to provide care to participants, as follow-up visits for trials give opportunities for continual medical examination and treatment. It is, nonetheless, morally praiseworthy to extend care to participants’ families and communities.

### Specifying the Content and Extent of Ancillary Care Obligations

The health capability paradigm establishes ancillary care obligations for external research funders, sponsors and investigators to clinical trial participants in LMICs. However, this duty cannot be the provision of unlimited ancillary care. There is an upper limit on the amount of ancillary care that investigators are expected to deliver in trial contexts. The health capability paradigm considers international clinical trials to be necessary to realize the ends of justice (Pratt *et al.*, 2012a). As noted by Merritt (2011), if achieving justice in global health requires the production of scientific results from clinical trials, parties’ ancillary care obligations cannot be so extensive that they usurp the necessary amount of resources (i.e. human, financial and physical) to complete a particular trial (Merritt, 2011).

The health capability paradigm prioritizes addressing those health conditions that are major contributors to the health gap between host communities and the optimal level. It further emphasizes efficiency (Ruger, 2010) and establishes obligations for global actors to assist where state systems are unable or choose not to deliver health care. Obligations are allocated on the basis of actors’ roles and technical skills (Table 2). International clinical researchers would, therefore, be required to meet only those ancillary care needs that they have the training and expertise to deal with. This may mean that the ancillary care offered in similar trials run by different research groups will vary. The paradigm additionally holds that cost-effectiveness and appropriateness be considered when determining what intervention(s) to provide for a particular health condition (Ruger, 2010).

From this, it may be inferred that international clinical researchers are obligated to provide ancillary care during trials for health conditions that meet the following selection criteria:

- they are major contributors to the health gap of host communities,
- local, state-run health care providers (public and private) are unable or choose not to offer treatment for the condition(s) and
- research personnel have the necessary expertise to treat the condition(s).

Where resources for ancillary care are limited, which will likely be the case for most international clinical research projects, they should be allocated to treatments for diseases that cause significant morbidity and mortality in host communities in order to promote the reduction of global health disparities. Such diseases can be reasonably expected to be encountered frequently. Where rare diseases with severe implications for health occur, if resources permit and the diseases meet the other selection criteria for ancillary care, international clinical researchers should address them as well. However, treating diseases that are not driving host communities’ health gap is secondary. Ancillary care is not a replacement for full health services. The ancillary care provided should also consist of interventions that are cost-effective and appropriate for the research population. The health capability paradigm further recognizes that other global actors have obligations to deliver health care to LMIC communities. If local medical non-governmental organizations (NGOs) are delivering care for certain conditions, the functional requirements principle does not demand researchers do so as well.

The health capability paradigm’s guidance regarding the content of ancillary care obligations is fairly consistent with the partial-entrustment and whole-person models, though differences do exist, particularly with respect to the scope of candidate ancillary care needs (Table 1). To advance global justice, identifying the scope of ancillary care needs requires a broad assessment of the health situation of host communities (i.e. what conditions are causing significant morbidity and mortality). The partial-entrustment model, in contrast, requires awareness of what health conditions are likely to be diagnosed through study-related tests.

**Table 1.** Different models’ articulation of the content of ancillary care obligations

	Health capability paradigm model	Duty of rescue component	Partial-entrustment model	Whole-person model
Scope of candidate ancillary care needs	Health condition is a major contributor to the health gap of host communities.	Health condition is severe and/or urgent.	Health condition is entrusted to researchers through consent process.	All health conditions encountered.
Criteria to determine whether a (strong) obligation exists to meet a candidate ancillary care need	**An absence of others able to meet that health need.** **Cost-effectiveness and appropriateness of available interventions.** **Researchers possess the expertise and technical capacity to meet the need safely.**	**An absence of others able to meet that health need.** **Ability to meet the need without incurring ‘inordinate’ costs.** **Researchers possess the expertise and technical capacity to meet the need safely.** Researchers’ freedom from competing obligations.	**Participants’ dependence on researchers (whether they lack other sources of help).** **Cost (money, personnel, study power).** Participants’ vulnerability (how badly off they would be if they did not receive help). Duration of researcher–participant relationship. Participants’ uncompensated risks and benefits.	**Participants’ dependence on researchers (whether they lack other sources of help).** **Cost (money, personnel, study power).** Participants’ vulnerability (how badly off they would be if they did not receive help). Duration of researcher–participant relationship. Participants’ uncompensated risks and benefits.

*Note:* Where there is alignment between two or more models, text is highlighted in bold.

## Empirical Research

### The Case Study

A case study was undertaken to determine the extent to which the ancillary care obligations supported by the health capability paradigm are capable of being upheld in practice and to identify the factors that make doing so feasible. It was intended to demonstrate what is possible, though not necessarily common practice in international clinical research. We selected SMRU and its ongoing vivax malaria treatment (VHX) trial as our case study because SMRU has been consciously designing its clinical trials to meet the health needs of its host community for the past 25 years (Cheah *et al.*, 2010). There was a high likelihood that one of its trials would involve the provision of ancillary care and provide us with data on how it was achieved. We describe both SMRU and its VHX trial below.

SMRU was established in 1985 as a field unit of the Mahidol-Oxford Tropical Medicine Research Unit. It is located on the Thai–Myanmar border in Mae Sot, Thailand and conducts its research with Karen and Myanmar refugees, migrants and displaced persons. Myanmar (Burma) has a long history of ethnic conflicts and political instability. The Karen, one of the largest ethnic groups in Myanmar and northern Thailand, has been engaged in armed rebellion against the Myanmar military forces since 1949. This resulted in population being displaced from eastern Myanmar to Thailand in 1984. Since 1995, there has also been a new influx of Myanmar refugees and migrants, including the Karen, coming to Thailand in search of work. Accordingly, we take the position that Thailand acquires a secondary obligation to enable these migrant workers and displaced persons to access its health system and to provide care to the Karen and Myanmar refugee population living in camps,^7^ with Myanmar retaining primary responsibility for ensuring the population’s health capabilities.^8^ However, neither Myanmar nor Thailand fully meets its obligations. Consequently, SMRU established clinics over a 15-year period to fill the health care gap for the border population. It functions as both a research unit and health care provider.

**Figure 1.**
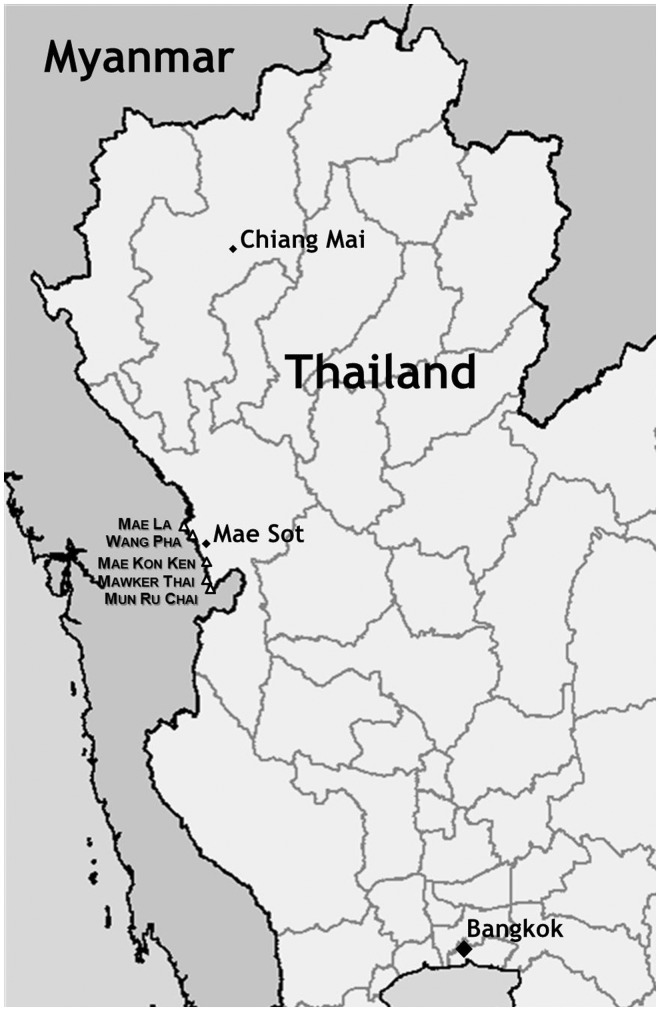
Map of the location of the SMRU office in Mae Sot, Thailand and the SMRU clinics along the Thai–Myanmar border.

The VHX trial seeks to describe the epidemiology and compare the efficacy of three treatments for vivax malaria—chloroquine/primaquine, chloroquine and artesunate (web reference: http://clinicaltrials.gov/ct2/show/NCT01074905). Trial sites are five SMRU clinics—Mae La, Wang Pha, Mawker Thai, Mun Ru Chai and Mae Kon Ken—located within 1 h’s drive of Mae Sot, Thailand (Figure 1). Across these five sites, there were roughly 410 VHX trial participants at the time of our research. Each was randomized into one of the three treatment groups and was completing the trial’s 1-year follow-up period. Funding for the trial is provided by Wellcome Trust.

### Research Methods

Case study methodology was selected because it enables exploration of *how* or *why* a complex social phenomena works and can bring out important contextual conditions (Yin, 2008). Data on SMRU’s VHX trial were collected using a triangulation approach that relied on a mix of qualitative research methods—in-depth interviews, direct observation and document analysis. Nineteen in-depth interviews were conducted with four types of VHX trial stakeholders—investigators (five interviews), Tak Province Border Community Ethics Advisory Board (T-CAB) members (four interviews), trial participants (eight interviews) and funder representatives (Wellcome Trust science portfolio advisors) (two interviews). Examples of interview questions are provided in Pratt *et al.* (2012b). Interview data were supplemented by direct observation at four of the five VHX trial sites over a 5-week period in March and April 2011 and by an examination of trial-related documents such as VHX trial participants’ case report forms (Pratt *et al.*, 2012b). At trial sites, we first observed for the standard examinations and treatments given to VHX trial participants. When this baseline was determined, we then observed for the provision of any additional examinations and treatments that deviated from this norm to identify ancillary care delivery. Case report forms included a form for Concomitant Medications, which listed all the medications a trial participant received for conditions other than vivax and falciparum. At four of the five trial sites, we randomly sampled 50 anonymized case report forms (200 in total) to generate a picture of what non-malarial conditions were treated during the trial.

All interviews were transcribed verbatim and translated from Burmese to English (where required). To ensure the accuracy of translation, two interviews that had been fully transcribed in Burmese were sent to a co-investigator who is fluent in Burmese and English to translate. We compared his translation of the interview transcript to that of our transcriber and found no significant discrepancies. Data were then analysed according to the principles of thematic analysis described in Braun and Clarke (2006), with co-coding performed independently by two researchers. Once themes were identified that pertained to the provision of ancillary care, we assessed whether the collated data extracts from each provided evidence that the VHX trial met the health capability paradigm’s requirements (Pratt *et al.*, 2012b). The results of that analysis are discussed below.

### Ancillary Care Provided During the VHX Trial

**Figure 2.**
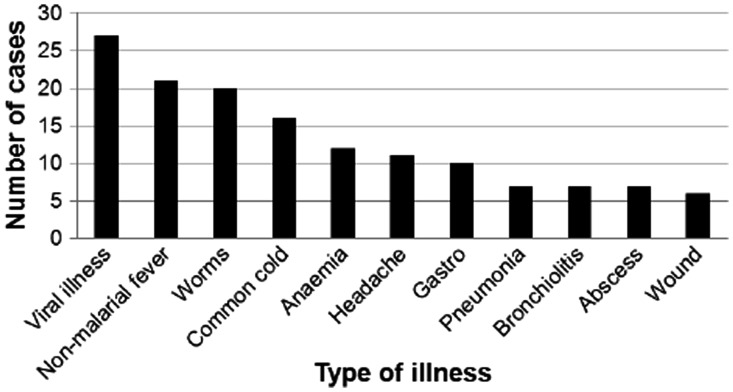
Most common conditions for which ancillary care is provided during the VHX trial as of April 2011 (based on analysis of 200 case report forms).

Case report form analysis demonstrates that, beyond vivax and falciparum malaria, VHX trial participants are treated for a wide variety of conditions (listed in Box 1). The most common conditions to be treated are viral illness, non-malarial fever, worms, common cold, anaemia, headaches and gastroenteritis (Figure 2). Viral illness, fever, colds and headaches are treated with paracetomol. Cases of worms are treated with mebendazole; gastroenteritis with aluminium hydroxide and anaemia with vitamin B complex, vitamin C, folic acid and ferrous sulphate. If necessary, trial participants are admitted to SMRU clinic in-patient departments. Case report form analysis indicates that two participants were admitted to Wang Pha Clinic and Mawker Thai Clinic in-patient departments, respectively, with conditions deemed to be unrelated to study drugs: scrub typhus and urinary tract infection. Of the eight trial participants who were interviewed, only one had needed care for a non-malarial health issue—an ulcer on his leg for which he was given treatment at each of his follow-up visits to Mae La Clinic for the VHX trial.

**Box 1. Conditions for which ancillary care is provided during the VHX trial**

1. Abscess

2. Amoebic dysentery

3. Anaemia

4. Bronchiolitis

5. Bronchitis

6. Chronic gastric ulcer

7. Common cold

8. Conjunctivitis

9. Dengue

10. Diarrhoea

11. External otitis

12. Fever (non-malarial)

13. Fungal infection

14. Gastroenteritis

15. Headache

16. Herpes simplex, STIs

17. Insomnia

18. Joint pain, arthritis

19. Kidney stone

20. Leptospirosis

21. Neuropathy

22. Non-severe pneumonia

23. Oral thrush

24. Otitis media

25. Pain

26. Pharyngitis

27. Scabies

28. Scrub typhus

29. Severe abdominal pain

30. Skin infection

31. Sty

32. Tonsillitis

33. Typhoid fever

34. Ulcer

35. Urinary tract infection

36. Viral illness

37. Worms

38. Wounds

The majority of ancillary care is provided by the SMRU medics, nurses and home visitors responsible for the day-to-day running of the VHX trial. These staff are recruited from the border population and trained in clinical care and research. Some have a background in health care before coming to SMRU, but others have less or no experience, as farming and wood-cutting are the main forms of employment in the rural areas of Myanmar bordering Tak province in Thailand. Staff training is administered through lectures, practical sessions and on-the-job training (e.g. daily patient rounds). At each trial site, three or four staff members are in-charge of collecting trial data and are supervised by VHX trial investigators, who are also the doctors in-charge at SMRU clinics. At Mae Kon Ken Clinic, for example, a nurse and home visitor are responsible for running the VHX study. For simple diagnoses and treatments, they provide clinical care to trial participants. For more complex cases, a senior nurse and/or out-patient department medics diagnose and treat trial participants. Clinic staff consult with the site doctor in-charge when complex cases and/or potential adverse events arise.

Where SMRU is unable to treat trial participants’ illnesses, site doctors in-charge refer patients to other health care providers and arrange for their transport.^9^ SMRU has relationships with the other health care providers on the Thai–Myanmar border. These include the Thai hospitals (Mae Sot Hospital, Phop Phra Hospital, Marymount Hospital), the Myanmar hospital KoKo (near Wang Pha Clinic), AMI Hospital (in Mae La camp) and Cynthia Maung’s Mae Tao Clinic. Aside from Mae Tao Clinic and AMI Hospital, the hospitals generally charge fees for patients, which SMRU often pays.^10^

### Selection of Ancillary Care in the VHX Trial

What ancillary care is provided during the VHX trial is determined by the treatments available in the SMRU clinics that serve as trial sites. If trial participants become sick with non-malarial illnesses, they are given the care available at the particular clinic that they attend for the trial.

Since a SMRU-run system was already in place to meet the health care needs of the border population, there was no need to liaise with local health care providers prior to the start of the VHX trial in order to identify trial participants’ ancillary care needs. The types of ancillary care to be offered were simple to identify, as the treatments available at trial sites (clinics) had already been established by senior SMRU doctors.

The criteria used to select the range of ancillary care provided as part of the VHX trial were effectively those that SMRU uses to determine what clinical care it offers at its clinics. These selection criteria are largely consistent with the selection criteria endorsed by the health capability paradigm. SMRU provides care for most *acute* illnesses experienced by the border population, including but not limited to those health conditions that cause significant morbidity and mortality. In doing so, it offers ancillary care beyond that which global justice requires.^11^ SMRU does not offer treatments for chronic illnesses such as cancer, Hepatitis C or diabetes. HIV treatment is only provided to TB patients, pregnant women and women following delivery in order to prevent mother-to-child transmission. As Investigator 01 explains

[i]t’s not because we don’t care. It’s because, first of all, they are few compared to other needs and they are very difficult to treat in the long term because of the commitment to see the patient on a regular basis. This population is relatively mobile, so it’s difficult to start the treatment and you never know whether months later the patient will be still there. We treat HIV but only in pregnant women and after their delivery or in TB patients, not because we don’t care about HIV while not in those two categories, but because we can’t afford.

Thus, the criteria that determine which conditions SMRU treats include there being a high number of cases in the border population, having a low treatment cost *and* having a finite treatment period. Treatment for chronic conditions is not offered primarily because of cost, the low prevalence of such conditions relative to infectious diseases and the mobility of the border population.

In keeping with the health capability paradigm, SMRU’s other ancillary care selection criteria relate to filling a health care gap and having the technical capacity to provide treatment for a condition. In the VHX trial, SMRU provides ancillary care for a wide range of conditions because the Thai and Myanmar health systems are largely inaccessible to the border population. SMRU offers care for conditions treated in Thai clinics and hospitals rather than care for only those conditions not addressed by the Thai health system. In doing so, SMRU recognizes that, while the Thai hospitals are in the vicinity, for refugees, illegal migrants and displaced persons, they are physically and financially difficult to access. Getting to Thai hospitals requires overcoming significant obstacles such as leaving Mae La camp, travelling 15–20 (or more) kilometres beyond the border and/or passing through military checkpoints intended to prevent Myanmar nationals from crossing into Thailand. Such barriers are not frequently overcome. Unlike SMRU clinics, Thai hospitals also charge patient fees. It should be said, however, that whenever a non-Thai patient, whether legally in Thailand or not, manages to present him/herself to a public hospital, s/he is provided with care. If the patient is too poor to pay, this care is free. Annually, the Thai public hospitals of Tak Province are millions of Thai baht short of their budget due to the extra cost of providing care to the non-Thai population.

Since the Thai and Myanmar health systems are largely inaccessible, SMRU migrant clinics, Wang Pha and Mawker Thai, offer health care for most infectious diseases free-of-charge. The situation in Mae La refugee camp is slightly different, as SMRU and AMI Hospital share health care provision responsibilities. SMRU provides antenatal, newborn and paediatric specialized care and treats cases of malaria. AMI Hospital provides care for the remainder of adult health problems free-of-charge. Mae Kon Ken Clinic is smaller than other SMRU clinics and treats mainly fever-related illnesses. All five SMRU clinics have a laboratory, out-patient department and in-patient department where patients can receive daily, free consultations and care. Mae La, Wang Pha and Mawker Thai clinics have antenatal care, delivery and special care baby unit facilities. Finally, SMRU provides ancillary care during the VHX trial for health conditions that Karen and Myanmar clinic staff have the skills and technical capacity to meet safely and that are relatively inexpensive to treat. At Mawker Thai Clinic, for example, this means that local staff generally do not perform lumbar punctures because there is not a sufficient volume of patients for them to maintain the skill level necessary to do this procedure safely.

### Ancillary Care Provision Creates Little Inequality in Health Care Access

The health capability paradigm identifies trial participants as the primary recipients of ancillary care obligations. However, as the paradigm supports providing ancillary care where there is an absence of others able to meet a health need, there is potential that its delivery will create inequalities in access to care between trial participants and non-participants. We recognize this issue and feel that it is important to consider how research groups might deal with it because it is highly desirable to avoid creating inequality within host communities. SMRU works to avoid generating this type of inequality and did not create it in the VHX trial, which is significant and worthy of discussion.

SMRU provides ancillary care to participants during trials and to non-participants on a continual basis. It is able to do so because it runs the clinics that serve as its research sites. The health care provided to non-participants can be considered ancillary care because the clinics would not have been set up had SMRU not wanted to do research in the area. The ancillary care provided to VHX trial participants is largely equivalent to the care otherwise provided to SMRU clinic patients, which means inequalities in access to health care are not generated between participants and non-participants. According to Investigator 01, ‘there is no different level of quality of health care because you are part of the study or you are not. It’s the same. It’s what we can provide to anybody.’ Investigator 02 concurs, stating ‘we treat the same as the out-patient, so they will get the same kind of medical care as the other patients who are not in the study.’ For Mae La, Mawker Thai and Wang Pha clinics, this generally holds true because they have the capacity to treat a wide variety of illnesses.

Mae Kon Ken Clinic, however, is only slightly larger than a dispensary and treats mainly fever-related illnesses. As a result, the provision of ancillary care to trial participants does create inequalities in health care access. Investigator 04 affirms

my intention is to not to divide between the OPD [out-patient department] and study but we cannot refuse or deny for their complaint in the study. Otherwise, they will looking for the drug outside and it can interfere with the study result. So that is why here is study cases get more health care. They receive more health care than [OPD patients], but not at the other clinics. Ideally, we have the equal health care system to the all patient.

During the VHX trial, patients at Mae Kon Ken Clinic receive less ancillary care than trial participants *and*, in general, they have less access to care compared with patients at other SMRU clinics. Investigator 04 suggests that creating some inequalities in access to care is necessary in order to ensure the validity of study results.

Ultimately, the VHX trial’s provision of care for non-fever-related illnesses does introduce an inequality in access to health care at one trial site. Even so, pre-existing inequalities in access to care between patients at Mae Kon Ken Clinic and other SMRU clinics are not exacerbated by the VHX trial. If anything, these inequalities are slightly reduced, with Mae Kon Ken trial participants being able to access more care than they otherwise would.

### Facilitating and Obstructive Factors

VHX trial investigators upheld their obligation to provide ancillary care to trial participants. This was strongly facilitated by SMRU’s long history of combining research and health care services with the active involvement of the border population (its staff). Since SMRU is already an established health care provider on the Thai–Myanmar border, trial investigators were able to utilize existing structures that cater to the health needs of the population from which trial participants were drawn. They did not need to organize to bring additional medical equipment or medicines (unrelated to the treatment of vivax) into the field. All that was needed to supply ancillary care to VHX trial participants was in place.

Combining a research unit and a medical NGO into a single organization is unusual. However, it was done to uphold a moral obligation identified by SMRU chief investigators to provide care to the border population. This obligation was seen to arise when research is performed in settings where the host population lacks access to a functional health system. As affirmed by Investigator 05, in such cases, the two roles—doing research and providing health services—cannot be dissociated,

[i]n the specific context, there are vulnerable populations such as the migrants and workers and the refugees, and displaced persons, as they’re called along the border. They didn’t have another source of health care, so we were morally obliged to, you know, you couldn’t just go in there and do research and ignore important past problems … So again it’s context specific, but I think there is a moral obligation.

This sentiment underlies the structure of SMRU and reflects the vision of the two men—Nick White and Francois Nosten—who established the research unit in 1985 and continue to run it today. They did not think it was ethical to simply go in and do research when the Karen and Myanmar border population lacked access to the Thai and Myanmar health systems. Consequently, SMRU set up health structures for the border population. These structures enable VHX trial investigators to efficiently and effectively fulfil the health capability paradigm’s requirements with respect to ancillary care provision.

Aside from delivery structures, financial support and human resources are necessary for the provision of ancillary care in international clinical research. With respect to the former, the selection of research funder is key. For the VHX trial, the cost of ancillary care is largely supported by Wellcome Trust. The Trust allocated funds for patient care that cover most non-trial-related medical expenses, as most health problems experienced by trial participants are inexpensive to treat. Investigator 05 notes

the Trust are pretty understanding. Their mandate is the research. I mean, they’re not there to give aid. Lot more money in the world for aid than there is for research, but they’re not narrow-minded about this. Some funders are more narrow-minded, but I can’t give you specific examples because we tend to avoid those funders.

Unlike research funders such as the US National Institutes of Health, which is restricted by government regulations, Wellcome Trust permits the use of its research money to pay for ancillary care. However, there are limits to Wellcome Trust’s support, as the allocation for patient care is a set amount. In cases where Wellcome Trust’s budget is insufficient, SMRU takes its moral obligation to provide a service to the border population quite seriously,

[i]f [a trial participant] got run over by a bus, trampled by an elephant, or had something rather weird, I suspect we wouldn’t have had an allocation for that. That’s the nature of these things. We’ll still look after these people obviously. So it might be that some funds for unusual problems might come from other pots within the SMRU budget. I mean we feel responsible, we can’t just say, sorry we didn’t think you we’re going to have that, so we’re not going to look after you.

VHX trial investigators are able to fulfil the framework’s requirements due to the clinical care capacity of the Karen and Myanmar staff at SMRU clinics. These staff are responsible for the day-to-day running of the VHX trial and have been trained as medics and nurses, which enables them to diagnose and treat non-study-related health conditions. Their clinical training is administered on a continual basis, with on-the-job training received during daily in-patient department rounds with SMRU doctors and training sessions delivered for different types of staff (e.g. midwives, medics, nurses and laboratory staff).

Obstacles to the provision of ancillary care do exist but are not a noticeable impediment in the VHX trial. These obstacles relate to funding and building the clinical capacity of Karen and Myanmar clinic staff. Sustaining SMRU’s service component has become more difficult over the years, as the number of clinics, staff and the population they serve have expanded. Although the cost of building new clinics is cheap, staff salaries are not and comprise 70 percent of SMRU’s budget. According to one investigator, ‘it’s a constant battle to try and get enough money to provide the service component because that’s a lot of staff and a lot of money for drugs and things.’ It is also becoming more difficult to pay for referrals that require expensive hospital care. Since SMRU is identified as a research organization rather than a medical NGO, it does not have ready access to certain avenues of funding open to humanitarian organizations. At present, SMRU health services are largely supported by grants from the European Union, Global Fund and UK Department for International Development.

VHX trial investigators suggest that there are difficulties inherent in building the clinical capacity of Karen and Myanmar individuals recruited to work at SMRU from the border population. This is likely to be the result of many factors including lack of familiarity with the requisite medical knowledge among these staff, their perceptions of their relationship with expatriate doctors in-charge and fear of doing the wrong thing. Comments were made concerning the reluctance of clinic staff to exercise initiative. Teaching abilities vary among expatriate doctors, who do not necessarily come to SMRU with experience in training people with limited education who have grown-up in a rather oppressive culture. Depending on the combination of doctor and staff, clinical capacity-building, particularly with respect to making diagnoses and treatment recommendations independently, can either be quite successful or a challenge.

### Fulfilment of Ancillary Care Obligations in the VHX Trial by External Stakeholders

Wellcome Trust and VHX trial investigators largely fulfil the health capability paradigm’s requirements for ancillary care (Table 2). The UK’s government has not enacted policies that require international clinical trials to provide ancillary care, but its laws do permit the use of research funding for non-study-related care, as the UK Medical Research Council does (Philpott *et al.*, 2010). Oxford University (sponsor) does not appear to have a significant role in SMRU health services, though it and the Mahidol-Oxford Tropical Medicine Research Unit do play a role in administering the grants that support these services. Nonetheless, it is difficult to envisage it being necessary for Oxford University to take on a role supporting the identification of the border population’s ancillary care needs. SMRU already has systems in place to capture this information. They include: regular conduct of epidemiological surveys, following prospective cohorts, clinic data collection systems and gathering information from local clinic staff.

**Table 2.** Ancillary care obligations supported by the health capability paradigm

Obligation bearer	Obligations of justice
National governments	• Enact regulations that require international clinical research to provide ancillary care. • Abolish policies and laws that impede international clinical research actors from meeting their ancillary care obligations.
Funders	• Fund the provision of ancillary care identified as ethically essential by trial investigators (in grant applications). • Abolish policies that restrict the use of research funding to provide ancillary care to trial participants.
Sponsors	• Support researchers to take the steps necessary to identify the ancillary care they have a duty to provide.
Researchers	• Identify the ancillary care needs that are ethically essential to address during a trial by, first, identifying illnesses with serious effects that are prevalent in host communities, second, determining if local health services or NGOs provide treatments for these conditions, third, determining whether trial personnel have the technical skills to treat the conditions and, fourth, identifying cost-effective and appropriate interventions for health conditions that meet criteria 1–3. • Provide ancillary care for those conditions to participants during the trial.

## Conclusions

This article has shown that a theory of justice—the health capability paradigm—can provide guidance on the provision of ancillary care in international clinical research that is capable of practical application. It has been noted that international research ethics guidelines generally do not include requirements for the provision of ancillary care (Participants in the 2006 Georgetown University Workshop on the Ancillary Care Obligations of Medical Researchers Working in Developing Countries, 2008). Our theoretical analysis suggests that obligations of justice demand the provision of ancillary care. Our empirical findings confirm that fulfilment of these obligations is feasible in LMIC settings where there is strong commitment to doing so from chief investigators and funders. There is no obvious reason why requirements for providing ancillary care should not be made part of international guidelines such as the *Declaration of Helsinki* (which is currently being revised). These requirements could retain some flexibility (e.g. allow for permissible exceptions), as there may be instances where ancillary care is not provided directly but the research is still ethical.

Our case study research further describes a strategy that has led to fulfilment of the ancillary care obligations required by the health capability paradigm in a context where trial participants could not access their state’s health system. Assuming a dual role as research unit and health care provider reflects an effective and pragmatic response to states’ (Thailand and Myanmar) unwillingness to fully respect their obligations of justice.^12^ SMRU, under the leadership of its chief investigators, has undertaken significant and sustained health care capacity-building on the Thai–Myanmar border, creating a health care system that supplements inaccessible state health systems. Over the years, SMRU has gradually expanded the number of clinics it runs and the services they offer in order to meet the health needs of, initially, the Karen and Myanmar refugee population and then the population of migrants and displaced persons living on the border. Investigator 01 states that

[f]or many years, we were known as a fever clinic, so people would come if they thought they had malaria, but if they had a broken arm, they would not come to us. But because of the increasing size of the population and that there are no other health structure in the area, except the Thai hospital, which is difficult for them to reach and costly, then we now have many more larger spectrum of conditions and diseases and we try to cope with everything.

Another investigator affirms: ‘Now we do the TB, delivery, neonatal, and immunisations, all those things, so it’s like the SMRU is filling the gap in this for the area.’ Nevertheless, this approach is not without its challenges, especially in relation to long-term sustainability of funding and building human resource capacity.

Ultimately, this case study takes the first step towards developing guidance on how one might fulfil ancillary care obligations in international clinical research. Other successful strategies need to be identified, as LMIC settings differ considerably. The context of the VHX trial is somewhat uncommon, as it involves refugee and migrant populations that fall entirely outside of state health systems. More frequently, international clinical research is performed in settings where state health systems exist but are under-resourced and offer limited care. VHX trial investigators think that SMRU’s general approach would work in this sort of environment, with the caveat that, rather than setting up clinics, researchers run studies through local clinics or partner with the district health department to do health research. However, other approaches may be employed in such settings in practice and these approaches need to be described in order to generate more comprehensive guidance on how ancillary care obligations might be met.

## Notes

1. The health capability paradigm is particularly suitable for the purpose of deriving such guidance because it has principles of justice that: (i) require the conduct of health research and research capacity strengthening and (ii) can be relied upon to establish specific obligations for external research actors from high-income countries to improve the health of individuals in LMICs.

2. The duty of rescue is an elementary moral principle. It affirms that everyone has a duty to help people who are in need and whom no one else is well-situated to help, provided that one can do so without serious sacrifice or risk (Merritt *et al.*, 2010). When researchers are the only agents who can help, the duty is theirs. The duty of rescue sets the baseline of what ancillary care is owed. It describes researchers’ minimum obligation to provide ancillary care that accrues simply by their being in the right place at the right time with the needed resources.

3. Entrustment refers to the fact that research participants automatically entrust certain aspects of their health into researchers’ care when they consent to join a study or clinical trial. Since a participant typically gives permission for a disease under study to be monitored, the scope of entrustment typically includes caring, as needed, for that disease, and following up on any clinically relevant information or diagnoses generated during the study.

4. The whole-person model does not provide an account of what it is about the researcher–participant relationship that gives rise to special ancillary care duties. It refuses to privilege any one attribute of the researcher–participant relationship as the basis for such duties (Merritt, 2011).

5. Although others have used the terms funder and sponsor interchangeably, we distinguish between the two in this article because, in international clinical research, they are often not the same entity. In the vivax malaria treatment trial that we discuss, for example, the funder was Wellcome Trust and the sponsor was Oxford University.

6. We recognize that some external research actors from high-income counties may have already internalized the norm of health equity.

7. Even though the United Nations’ refugee convention does not classify all Karen and Myanmar migrants and displaced persons as being owed formal legal obligations by Thailand, we take the position that Thailand has a *moral* obligation to provide health care to not only refugees but also migrants and displaced persons on the border.

8. We recognize that the political climate is shifting in Myanmar, following the elections in November 2011. However, so far, this has not resulted in significant changes on the Thai–Myanmar border. As a result, Thailand maintains its secondary obligation to provide health care to the border population.

9. Case report form analysis shows that only one VHX trial participant (of the 200 whose case report forms were examined) was referred for care during the study, first to AMI Hospital and then s/he was transferred to Mae Sot Hospital for severe abdominal pain. As this was a severe adverse event, it is considered study-related care rather than ancillary care provision.

10. Registered migrant workers are entitled to some health care from Thai hospitals if they purchase health insurance, but this does not cover treatment during research experiments.

11. VHX trial participants receive ancillary care for non-severe illnesses that are inexpensive and simple to treat. Although the health capability paradigm does not require treatment to be given for such illnesses, this does not mean that it disallows such actions. Clearly, there is flexibility to engage in such morally praiseworthy behaviour.

12. The strategy would also be applicable where states are unable to fulfil their obligations, particularly in areas where there is a complete lack of state health structures. Where health structures exist but are under-resourced, the health capability paradigm would call for research groups to work through state health systems and to strengthen their capacity instead of setting up new clinics. (To do this, research groups could still assume a dual function.)
